# Transcriptome sequencing of Crucihimalaya himalaica (Brassicaceae) reveals how Arabidopsis close relative adapt to the Qinghai-Tibet Plateau

**DOI:** 10.1038/srep21729

**Published:** 2016-02-24

**Authors:** Qin Qiao, Qia Wang, Xi Han, Yanlong Guan, Hang Sun, Yang Zhong, Jinling Huang, Ticao Zhang

**Affiliations:** 1 School of Agriculture, Yunnan University, Kunming 650091, China; 2 Key Laboratory for Plant Diversity and Biogeography of East Asia, Kunming Institute of Botany, Chinese Academy of Sciences, Kunming 650201, China; 3 University of Chinese Academy of Sciences, Beijing, 10049, China; 4 Key Laboratory for Biodiversity Science and Ecological Engineering, School of Life Sciences, Fudan University, Shanghai 200433, China; 5 Department of Biology, East Carolina University, Greenville, NC 27858, USA

## Abstract

The extreme environment of the Qinghai-Tibet Plateau (QTP) provides an ideal natural laboratory for studies on adaptive evolution. Few genome/transcriptome based studies have been conducted on how plants adapt to the environments of QTP compared to numerous studies on vertebrates. *Crucihimalaya himalaica* is a close relative of *Arabidopsis* with typical QTP distribution, and is hoped to be a new model system to study speciation and ecological adaptation in extreme environment. In this study, we *de novo* generated a transcriptome sequence of *C*. *himalaica*, with a total of 49,438 unigenes. Compared to five relatives, 10,487 orthogroups were shared by all six species, and 4,286 orthogroups contain putative single copy gene. Further analysis identified 487 extremely significantly positively selected genes (PSGs) in *C*. *himalaica* transcriptome. Theses PSGs were enriched in functions related to specific adaptation traits, such as response to radiation, DNA repair, nitrogen metabolism, and stabilization of membrane. These functions are responsible for the adaptation of *C*. *himalaica* to the high radiation, soil depletion and low temperature environments on QTP. Our findings indicate that *C*. *himalaica* has evolved complex strategies for adapting to the extreme environments on QTP and provide novel insights into genetic mechanisms of highland adaptation in plants.

Extreme environments provide an ideal natural laboratory for studies on adaptive evolution. The Qinghai-Tibet Plateau (QTP) is the highest (above 4,000 meters of average elevation) and largest young plateau in the world, which has low temperature, low oxygen, poor soils, and strong ultraviolet (UV) radiation environments. Understanding how organisms adapt to these various extreme environments can make a significant contribution to evolutionary ecology. Recently, research on the adaptive genetic mechanism of non-model organisms based on genome-wide scale can be conducted using the next generation sequencing technology^1^. Previous genome-wide studies on adaptive evolution on this region have focused mainly on humans and vertebrates^2^,^3^,^4^,^5^,^6^,^7^,^8^. These studies revealed that genes involved in hypoxia response, energy metabolism, and skeletal development were under positive selection and rapidly evolving. However, little genome/transcriptome-based research has been devoted to plants in this region.

Relatives of *Arabidopsis thaliana* are important model systems to study evolutionary ecology and comparative genomics^9^,^10^. Several studies have been conducted on the evolution of mating systems in *Capsella*^11^,^12^, life history form in *A*. *lyrata* and *Capsella rubella*^13^, as well as adaptation to serpentinite soils in *A*. *lyrata*^14^ and external climates in *A*. *halleri*^15^. Notably, there are other close relatives of *Arabidopsis*, which not only occupy distinctive niche, but also are of ecological importance. *Crucihimalaya himalaica* (Edgeworth) Al-Shehbaz has been known as *A*. *rupestris* Edgeworth, *A*. *himalaica* (Edgeworth) O. E. Schulz, *Arabis brevicaulis* Jafri, and *A*. *brevicaulis* (Jafri) Jafri^16^. This species is a diploid (2n = 16) with a relatively small genome size of 319 Mb^17^. It usually grows in rocky hillsides, sandy slopes, alpine meadows, and screes in areas around Himalayas, e.g. QTP.

Previous reviews suggested that *Crucihimalaya* is a specialized group in Himalayas derived from the *Arabidopsis* genus in very late geological history^18^,^19^. However, recent molecular phylogenetic result showed that *Crucihimalaya*, *Capsella*, *Arabidopsis* and several genera constitute a paraphyly^20^. Dated phylogenies also indicated that the genus *Crucihimalaya* origin in about 5.2 Mya, and that *C*. *himalaica* split from *C*. *lasiocarpa* in about 3.56 Mya^20^, which is in accordance with the time of QTP rapid uplift, 3.6 Mya^21^. Conceivably, this species must have undergone significant genetic changes to adapt to stress factors following the rapid uplift of QTP. Therefore, *C*. *himalaica* is hoped to be a new model system to study speciation and ecological adaptation in extreme environments.

Transcriptome analysis (RNA-seq) provides a rapid and effective approach to obtain massive protein-coding genes, which can be used for understanding ecological, comparative and evolutionary genomics questions for non-model organisms^22^,^23^. In this study, we performed RNA-seq to obtain most transcript sequences of *C*. *himalaica*. Subsequently, positively selected genes (PSGs) in *C*. *himalaica* related to environmental adaptation were identified by comparative genomics with closely related species whose genome have been sequenced. We aim to reveal how this *Arabidopsis* relative adapts to the complicated extreme environments on QTP at genome/transcriptome level.

## Results

### Transcriptome assembly and annotation

In this study, we generated 132.29 million clean reads and 16.52 Gb of RNA-seq data after quality filtering (Supplementary Table S1). The clean data were submitted to the NCBI Sequence Reads Archive (SRA) database (no. SRR3138110). *De novo* assembly of these high-quality reads generated 66,084 transcripts and 49,438 unigenes (the longest transcript in one gene) (Table 1). The unigenes are 896 bp on average with an N50 length of 1,641 bp.

**Figure 1:**
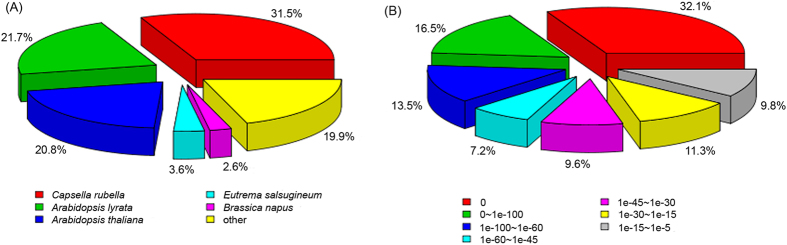
Species classification (**A**) and e-value distribution (**B**) of the unigenes of *C*. *himalaica* annotated to NCBI Nr database.

There are 29,760 (60.19%) and 33,667 (68.09%) unigenes identified homologs in Nr and Nt databases on the basis of similarity, respectively. A total of 39,189 (79.26%) unigenes were successfully annotated using at least one database (a total of seven databases, see methods) with a significant match (e-value < 10^−5^) (Supplementary Table S2). The top species classification hits for *C*. *himalaica* in the Nr database are *Capsella rubella*, *A*. *lyrata*, and *A*. *thaliana* (Fig. 1A), which all belong to Brassicaceae. The e-values are very significant, which mostly close to zero (32.1%) and 0∼1e–100 (16.5%) (Fig. 1B), suggesting that most unigenes of *C*. *himalaica* have very similar homologs in above relatives.

### Orthogroups identified and positive selection analysis

**Figure 2:**
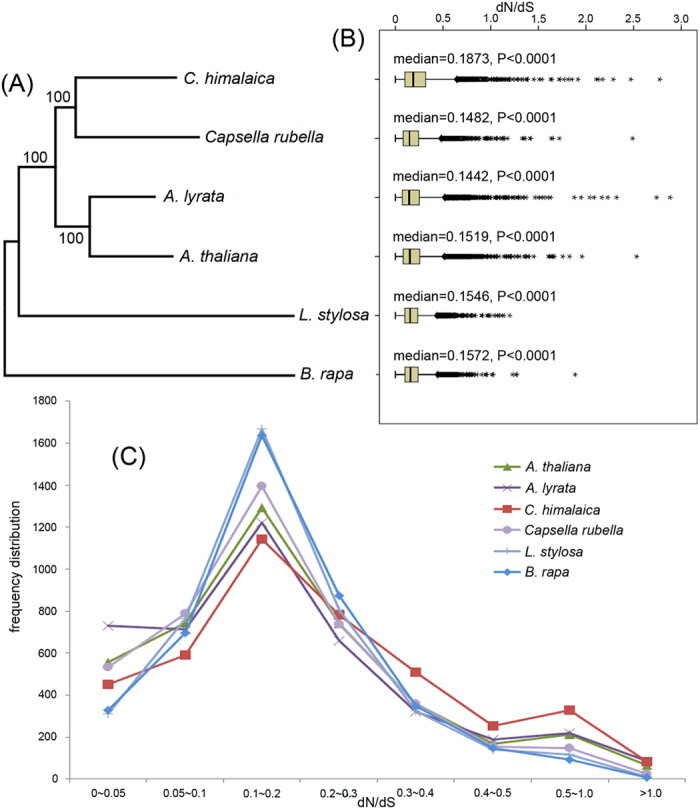
Phylogenetic relationships and dN/dS ratios distribution of *C*. *himalaica* and its relatives. (**A**) Phylogenetic tree derived from concatenated all orthologs (1,506,379 amino acids) of six species. (**B**) Boxplots of dN/dS ratios for each species. The median dN/dS ratio and significances of the deviations using Wilcoxon rank sum test are also showed in the boxplots. (**C**) Number of orthologs with given dN/dS ratios for the six species.

In this study, a total of 22,572 orthogroups were detected, including 152,769 genes. Among these, 10,487 orthogroups were shared by all six species, and 4,286 orthogroups contained putative one-to-one single copy genes. To further confirm the phylogenetic position of *C*. *himalaica*, we generated a phylogenetic tree based on 1,506,379 amino acid sequences of the trimmed and concatenated 4,286 single copy gene alignments from six species (Fig. 2A). The phylogenic tree showed that *C*. *himalaica* was closely related to C*apsella rubella*, and then clustered with the clade of *A*. *lyrata* and *A*. *thaliana*. The placement of *L*. *stylosa* was outside of these two clades with *B*. *rapa* as outgroup.

We also estimated the substitution rates for each orthogroups by free-ratio model in PAML, which allows an independent dN/dS ratio for each branch^24^. For each species, the dN/dS ratio major concentrated in 0.1–0.2 (Fig. 2B,C). The median of dN/dS ratio in *C*. *himalaica* (0.1873) across all the orthologs was significantly larger than that of other relatives (0.1442–0.1572) (Fig. 2B, Wilcoxon rank sum test, p < 0.0001). The frequency distribution of dN/dS ratios evidently showed that *C*. *himalaica* (1172 genes) has more genes with elevated dN/dS ratios (dN/dS > 0.3) than others (592–815 genes) (Fig. 2C).

For 4,286 single copy orthogroups, the branch-site model of the PAML 4 package^24^ was used to detect genes with signals of positive selection. As a result, 1,444 genes possibly under positive selection were identified in the *C*. *himalaica* genome (ω > 1); of these genes, 598 showed significant evidence of positive selection (P-value < 0.05), and 487 were undergo extremely significant positive selection (P-value < 0.01) (Supplementary Table S3).

**Figure 3:**
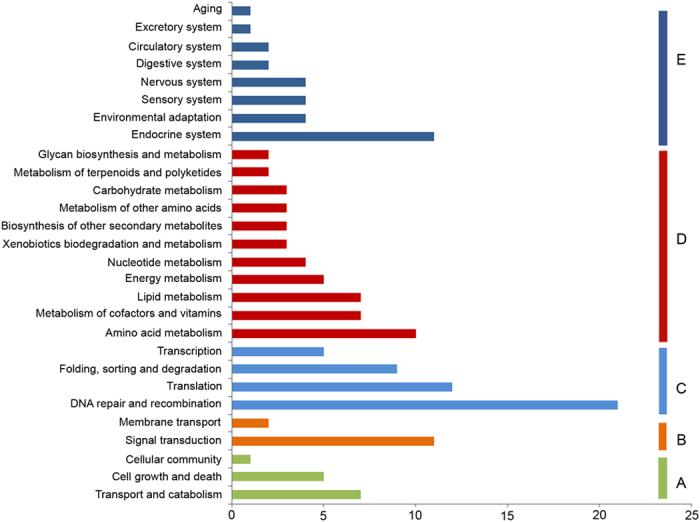
Distribution of KEGG classification of PSGs in *C*. *himalaica*. (**A**) Cellular Processes; (**B**) Environmental Information Processing; (**C**) Genetic Information Processing; (**D**) Metabolism; (**E**) Organismal Systems.

**Figure 4:**
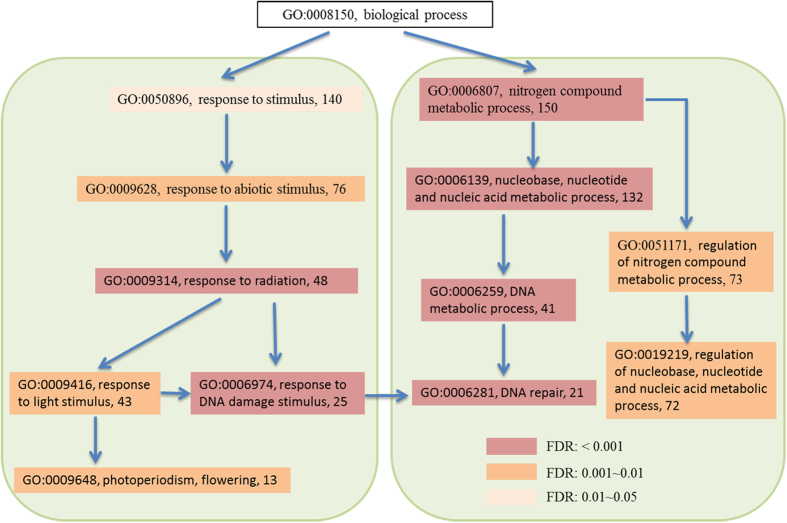
GO enrichment of PSGs related to ecological adaptation in *C*. *himalaica*. The Arabic numbers show the enriched number of PSGs in each term.

We also conducted KEGG (Fig. 3, Supplementary Table S4) and GO (Fig. 4, Supplementary Table S5) functional classification for these PSGs (P-value < 0.01) in *C*. *himalaica*. The distribution of KEGG classification of PSGs showed that in all annotated categories, metabolic processes have the most hits, such as amino acid metabolism (10 PSGs), cofactors and vitamins metabolism (7 PSGs), lipid metabolism (7 PSGs), and energy metabolism (5 PSGs) (Fig. 3). Beyond that, another predominant hit was DNA repair and recombination, which included 21 PSGs. It contains several DNA repair pathways, such as mismatch repair (6 PSGs), nucleotide excision repair (5 PSGs), base excision repair (3 PSGs) and homologous recombination (2 PSGs) (Supplementary Table S4).

In addition, GO annotation also showed that many PSGs are related to specific adaptation traits, including nitrogen compound metabolic process (150 PSGs), regulation of nitrogen metabolic (73 PSGs), response to radiation (48 PSGs), DNA repair (21 PSGs), and photoperiodism, flowering (13 PSGs) (Fig. 4). In the following paragraphs, we will discuss the important roles of these PSGs in the adaptation of *C*. *himalaica* to the various extreme environments in QTP, respectively.

## Discussion

Our phylogenetic analysis based on genomic level highly supported that *C*. *himalaica* was most closed to C*apsella* rather than *Arabidopsis*. The relationship among these species agrees with previous study^20^. Although previous reviews suggested that *Crucihimalaya* is a specialized group in Himalayas derived from the *Arabidopsis* genus^18^,^19^. Our result indicates that it should be derived from the common ancestor of the *Arabidopsis* lineage and *Crucihimalaya* lineage. In fact, *C*. *himalaica* grouped with *C*. *rubella*, but not *A*. *thaliana*, is conflict with morphological data, especially the principal character for taxonomy (dehiscent siliques, linear fruits in *Crucihimalaya* & *Arabidopsis* vs. dehiscent silicles, obdeltoid fruits in *Capsella*). Maybe that is why *Crucihimalaya* was classified into *Arabidopsis* in previous taxonomic system. This conflict between the morphological and molecular result highlights the need for further studies on the intrinsic mechanism of speciation.

Orthologs are genes that have evolved from a common ancestral gene via speciation^25^. To investigate the selective pressures at the branch level in *C*. *himalaica* and its relatives, we estimated the substitution rates for each orthogroups. The median of dN/dS ratio in *C*. *himalaica* was significantly larger than that of other relatives, which strongly supported the accelerated evolution in *C*. *himalaica* after splitting from its ancestor lineage. Furthermore, the frequency distribution of dN/dS ratios evidently showed that *C*. *himalaica* has more genes with elevated dN/dS ratios than other five species. These accelerated evolution of genes is often driven by positive selection or relaxed selection pressure. Combined with results of branch-site model, which revealed that many functional genes in *C*. *himalaica* undergo positive selection in extreme environment of QTP, we speculated that the evolutionary rate increased in *C*. *himalaica* are due to positive selection rather than relaxation of select.

There are very high light radiation on QTP, especially the UV-B radiation during the summer (approximately 65 kJ/m^2^) is among the highest worldwide^26^. The light radiation can influence plant growth and development, such as photoperiodism, flowering, and DNA damage. Our results showed that as many as 43 PSGs in *C*. *himalaica* were annotated as response to light stimulus according to GO category, among which, 13 PSGs in the *C*. *himalaica* genome were involved in photoperiodism, flowering process (Fig. 4, Supplementary Table S5). Among these, the gene encoding early flowering 6 protein (lysine-specific demethylase, ELF6) is a repressor in the photoperiodic flowering pathway and its loss-of-function mutation causes early flowering^27^. Similarly, another gene encoding sensitive to freezing 6 protein (mediator of RNA polymerase II transcription subunit 16), plays an important role in regulation of the circadian clock and in the control of flowering time^28^. In the short growing season condition of QTP, flowering time is particularly critical and affect both the life cycles and reproductive success of alpine flora^29^,^30^. *C*. *himalaica* starts flowering relatively early (April), and has a very long duration of the flowering period (from April to September). This pattern suggests that *C*. *himalaica* has evolved specific reproductive strategies via positively selected more than a dozen genes associated with photoperiodism and flowering process as adaptation to the short growing season in the harsh environments of QTP.

In addition, highly energetic UV radiation causes direct damage to DNA, RNA, and proteins^31^. Our results showed that 25 PSGs were involved in response to DNA damage stimulus (Fig. 4, Supplementary Table S5). This result was consistent with 21 PSGs enriched in DNA repair pathway (Fig. 4, Table 2). As an essential system for correcting UV-induced DNA damage, nucleotide excision repair (NER) play more important roles in UV-resistance for plants^32^. It is worth to mention that several essential genes participating in NER process showed positive selection. Such as gene encoding DNA excision repair protein ERCC-1 (or ultraviolet hypersensitive 7), which has been reported to function as a part of a structure-specific endonuclease that cleaves 5′ to UV photoproducts in DNA^33^. And gene encoding DNA damage-binding protein 2, which forms a complex with DDB1 to recognize damaged DNA and initiation of NER process after exposure to UV light^34^. Another gene, encoding TFIIH protein, is an essential transcription initiation factor that is also pivotal for NER^35^. Besides NER, other DNA repair mechanism, such as base excision repair (BER), mismatch excision repair (MMR) and homologous recombination (HR) also participated in response to light radiation and other external environment stimulation in QTP (Table 2). Accordingly, we found PSGs encoding DNA repair protein XRCC4, DNA mismatch repair protein MUTS2, MUTS protein homolog 2/5 (MSH2/5), MUTL protein homolog 3 (MLH3), DNA repair and recombination protein RAD54, and DNA damage repair/toleration protein DRT111 (Supplementary Table S3). KEGG enrichment showed that these genes covered almost all aspects of the DNA repair mechanism (Supplementary Table S4), suggesting that *C*. *himalaica* has evolved an integrated DNA repair mechanism to adapt to the harsh habitat following the uplift of QTP.

Nitrogen compound metabolism is the basic physiological processes, which can generate components of cells (e.g. nucleic acids and proteins) as well as convert sugars and proteins into energy. Moreover, nitrogen compound metabolism is even more important for *C*. *himalaica*, because nutrient deficiency, especially nitrogen deficiency is a typical feature of the sandy soil habitats for its living^36^,^37^. It means nitrogen assimilation under low-nitrogen condition is also a challenge for *C*. *himalaica* living in QTP. From our results, it was evident that as many as 150 PSGs involved in nitrogen compound metabolism in *C*. *himalaica*, which included GO categories associated with DNA metabolic process (41genes) and regulation of nitrogen compound metabolic process (73 genes) (Fig. 4, Supplementary Table S5). The latter included many transcription factors involved in the nitrogen assimilation process, including the primary assimilation of ammonia to carbon skeletons to biosynthesize amino acids and other organic compounds. Remarkably, three members of DOF (DNA BINDING WITH ONE FINGER) family, a class of zinc finger domain TFs, showed significant positive selection. DOF factors could play an important role in nitrogen regulation, which was supported by the fact that Dof1 over-expressed in *Arabidopsis* promote the nitrogen assimilation, thus improved plant growth under low-nitrogen conditions^38^. Moreover, a PSG encoding GATA zinc finger protein homolog NTL10, also has been reported activates the expression of nitrogen-catabolic enzymes during conditions of nitrogen limitation^39^. The functions of these PSGs suggested that *C. himalaica* may have adaptively sped up evolutionary rates of genes involved in nitrogen metabolic regulation to better adapt to the nitrogen limitation in its habitat, which seems to be the driving force behind rapid evolution of these genes.

Low temperatures and rapid changes of temperature are also prevalent features of QTP. Organisms that live in this alpine ecosystem face various growth-related challenges from low temperatures, such as reduced fluidity of lipid membranes^40^. The lipid bilayers transmit external signals to its interior as well as protect the integrality of various organelle membranes in unfavorable environments. Our GO category results indicated that 22 genes related to lipid biosynthesis were under significant positive selection, including 14 PSGs genes involved in phospholipid biosynthetic process and 5 PSGs in glycerolipid biosynthetic process (Supplementary Table S3). Among these, gene encoding lipid-A-disaccharide synthase with function of condense the lipid A, could play a structural role by stabilizing the plasma membrane lipid bilayer in plants, and involved in signal transduction or plant defense responses^41^. Other PSGs, such as gene encoding Phosphatidyl-N-methylethanolamine N-methyltransferase, was reported to produce the abundant membrane lipid phosphatidylcholine^42^. Previous findings also showed that the degree of unsaturated lipids remains unchanged in *C*. *himalaica* under rapid temperature changes^43^, suggesting that the remodeling of membrane lipids might protect membranes against frequent temperature changes. Based on above results, we speculate that these membranes lipids and proteins might play major roles in maintaining membrane integrity under low temperatures and rapid changes of temperature conditions.

## Conclusion

Organisms that live in QTP face a variety of external stress from their harsh environments. As such, it is often believed that these organisms have undergone a series of adaptive evolutionary changes. Low oxygen was reported as the most challenge for animals living in high-altitude, thus several genes response to hypoxia showed signature of positive selection in different species^2^,^3^,^4^,^5^,^6^,^7^,^8^. Unlike animals, plants as sessile organisms are constantly exposed to these stresses in QTP. It is conceivable that they have developed more sophisticate mechanisms to protect themselves. Our results of transcriptome sequence of *C*. *himalaica* and evolutionary genomic analysis provided evidence for this belief. In this study, we identified 487 significantly PSGs in transcriptome sequences of *C*. *himalaica*. Functions of these PSGs elucidate that they potentially possess specific traits of adaptive significance, such as response to radiation, DNA repair, membrane stabilization and organic metabolism. These functions are likely responsible for the adaptation of *C*. *himalaica* to the high radiation, low temperature and soil depletion environments on QTP. Due to the RNA-seq technology is difficult to obtain the entire and full length transcripts, further studies are required. Nevertheless, our findings indicate that sophisticated genetic mechanisms have evolved in *C*. *himalaica* to survive the harsh conditions in QTP.

## Materials and Methods

### Sample collection and transcriptome sequencing

The original seedlings of *C*. *himalaica* were sampled in August 24, 2013 in Xiangcheng County (alt. 4,104 m, 29°02′47″ N, 99°42′55″ E) of QTP. These seedlings from the same individual were cultivated in greenhouse in Kunming Institute of Botany. In order to obtain more expression transcripts, two developmental stages (15 days, 30 days) of different organs (leaves, stems, and roots) were sampled and stored at −80 °C.

High quality total RNA was extracted using the TRIZOL reagent (Sigma Aldrich) following the manufacturer’s instructions. A total amount of 3 μg RNA per developmental stage was used as input material for the RNA sample preparations. Sequencing libraries were generated using NEBNext Ultra™ RNA Library Prep Kit for Illumina Inc. (NEB, USA) following manufacturer’s recommendations. The clustering of the index-coded samples was performed on a cBot Cluster Generation System using TruSeq PE Cluster Kit v3-cBot-HS (Illumina Inc.). After cluster generation, the library preparations were sequenced on an Illumina Hiseq 2500 platform and paired-end reads were generated. The whole step of library construction and Illumina sequencing was performed at Novogene Bioinformatics Technology Co., Ltd (Beijing, China).

### *De novo* assembly and functional annotation

Clean data (clean reads) were obtained by removing reads containing adapter, reads containing ploy-N and low quality reads from raw data. At the same time, Q20, Q30, GC-content and sequence duplication level of the clean data were calculated. The sequenced left files (read1 files) from two samples were pooled into one big left.fq file, and right files (read2 files) into one big right.fq file. Transcriptome assembly was accomplished based on the left.fq and right.fq using Trinity program (trinityrnaseq_r20140413) with minimum k-mer coverage set to 2 and all other parameters set by default^44^.

Functional annotations of all assembled unigenes were conducted by searching against the following databases: NCBI non-redundant protein (Nr), NCBI non-redundant nucleotide (Nt), Protein family (Pfam), Clusters of Orthologous Groups of proteins (KOG), Swiss-Prot protein (Swiss-Prot), KEGG Ortholog database (KO), and Gene Ontology database (GO).

### Orthologous genes identified and phylogenetic analysis

Based on previous studies in Brassicaceae^10^,^20^, we selected genomes of five relatives (*A*. *thaliana*, *A*. *lyrata*, *Capsella rubella*, *Leavenworthia alabamica* and *Brassica rapa*) and *C*. *himalaica* to identify orthologs. Furthermore, to define a set of conserved genes for cross-taxa comparison, we used OrthoMCL software^45^ to identify homologous gene clusters (orthogroups) among the six genomes. Genes with lengths less than 50 amino acids were excluded. OrthoMCL was run with an e-value cut-off of 1e-15 and an inflation parameter of 2.0 due to the close genetic relationship between six relatives.

Orthologroups with only single copy genes (one-to-one orthologs) that were shared by all six genomes were retained for further analysis. Each orthogroups was aligned using MUSCLE v3.8.31^46^ with default parameters. The poorly aligned regions were further strictly trimmed by using the trimAl v1.4 software^47^ with the parameter “-gt 0.8 -st 0.001”. Alignments of all orthogroups were concatenated by our python script. Then maximum likelihood (ML) trees were generated using RAxML v7.0.4^48^ with PROTCATJTT model, the maximum likelihood criteria.

### Positive selection analysis

In the positive selection analysis, only above single copy genes were considered. To calculate the nonsynonymous (Ka) and synonymous (Ks) substitution rates between pairs of orthogroups, above amino acid alignments were reverse-translated to the corresponding codon-based nucleotide alignments by PAL2NAL^49^. For each alignment, a gene tree was constructed by RAxML^48^ using GTR + GAMMA model.

To estimate lineage-specific evolutionary rates for each branch of the six species, the codeml program in the PAML 4 package^24^ with the free-ratio model (model = 1) was run on each orthogroups. We conducted the boxplot analysis using the dN/dS ratio derived from free-ratio model results and filtered dS >3 or dN/dS >3. Significances of the deviations from the median dN/dS ratio between six species branches were detected using Wilcoxon rank sum test. We also established frequency distribution plots of all dN/dS ratios of six species.

To increase the power of our tests for positive selection, we applied the improved branch-site model^50^ implemented in codeml program^24^ to estimate the dN/dS substitution rates (ω value). We also deleted all gaps (clean data = 1) from the alignments to lower the effect of ambiguous bases on the inference of positive selection. A foreground branch was specified as the clade of *C*. *himalaica*. A significant likelihood ratio test (LRT) was conducted to determine whether positive selection is operating in the foreground branch. In this study, the extremely significant positively selected genes (PSGs) were inferred if the P-value was less than 0.01.

For each PSG in *C*. *himalaica*, functional information was inferred based on its ortholog in *A*. *thaliana*. Gene Ontology (GO) enrichment analyses of PSGs were conducted using web-based agriGO (bioinfo.cau.edu.cn/agriGO)^51^ with singular enrichment analysis (SEA) method and TAIR10 database. The KOBAS software^52^ was also used to test the statistical enrichment of PSGs in Kyoto Encyclopedia of Genes and Genomes (KEGG) pathways^53^.

## Additional Information

**How to cite this article**: Qiao, Q. *et al.* Transcriptome sequencing of Crucihimalaya himalaica (Brassicaceae) reveals how Arabidopsis close relative adapt to the Qinghai-Tibet Plateau. *Sci. Rep.* **6**, 21729; doi: 10.1038/srep21729 (2016).

## Supplementary Material

Supplementary Table S1

Supplementary Table S2

Supplementary Table S3

Supplementary Table S4

Supplementary Table S5

Supplementary Figure S1

Supplementary Figure S2

Supplementary Figure S3

## Figures and Tables

**Table 1:** Statistics of assembled data.

Name	200–500 bp	500–1 k bp	1 k–2 kbp	>2 kbp	Total	Min Length	Mean Length	Max Length	N50	N90
transcripts	28189	12098	14649	11148	66084	201	1117	16855	1917	440
unigenes	26001	9068	8663	5706	49438	201	896	16855	1641	329

**Table 2:** List of PSGs in DNA repair pathway in *C. himalaica*.

DNA repairing mechanisms	Gene name	Orthologs in *A. thaliana*
Base excision repair	NTH1; Endonuclease III homolog 1, DNA glycosylases	AT2G31450
	APEX2; Apurinic-apyrimidinic endonuclease 2	AT4G36050
	DPOD2; DNA polymerase delta subunit 2	AT2G42120
Nucleotide excision repair	DDB2; UV-damaged DNA -binding protein 2	AT5G58760
	RAD7; DNA repair protein RAD7	AT5G21900
	TFIIH3; transcription initiation factor TFIIH subunit 3	AT1G18340
	TFIIH4; transcription initiation factor TFIIH subunit 4	AT4G17020
	ERCC1; DNA excision repair protein ERCC-1	AT3G05210
Mismatch excision repair	MSH3; DNA mismatch repair protein MSH3, MutS protein homolog 3	AT4G25540
	MSH7; DNA mismatch repair protein MSH7, MutS protein homolog 7	AT3G24495
	mutS2; DNA mismatch repair protein MutS2	AT1G65070
	MLH3; DNA mismatch repair protein MLH3, MutL protein homolog 3	AT4G35520
	EXO1; 5′-3′ exonuclease family protein	AT1G18090
Homologous recombination	RQSIM; ATP-dependent DNA helicase Q-like SIM	AT5G27680
	RQL3; ATP-dependent DNA helicase Q-like 3, bloom syndrome protein	AT4G35740
	AP5Z1; AP-5 complex subunit zeta-1	AT3G15160
	XRCC5; ATP-dependent DNA helicase 2 subunit	AT1G48050
	XRCC4; DNA-repair protein XRCC4	AT3G23100
	FANCM; fanconi anemia group M protein	AT1G35530
	FAN1; fanconi-associated nuclease 1 homolog	AT1G48360
Other factors with DNA repair function	DCLRE1A; DNA cross-link repair 1A protein	AT2G45700
