# Supplementary material for: Transcriptome sequencing of Crucihimalaya himalaica (Brassicaceae) reveals how Arabidopsis close relative adapt to the Qinghai-Tibet Plateau
Source: Sci Rep. 2016 Feb 24;6:21729. doi: 10.1038/srep21729 (PMC4764839; doi:10.1038/srep21729)
Supplement: Supplementary Figure S3 [file srep21729-s8.doc]

**Transcriptome sequencing of** ***Crucihimalaya himalaica* (Brassicaceae) reveals how *Arabidopsis* relatives adapt to Qinghai-Tibet Plateau**

Qin Qiao ^1^, Qia Wang ^2^, Xi Han ^2^, Yanlong Guan ^2^, Hang Sun ^2^, Yang Zhong ^3^, Jinling Huang ^4^, Ticao Zhang ^2*^

A
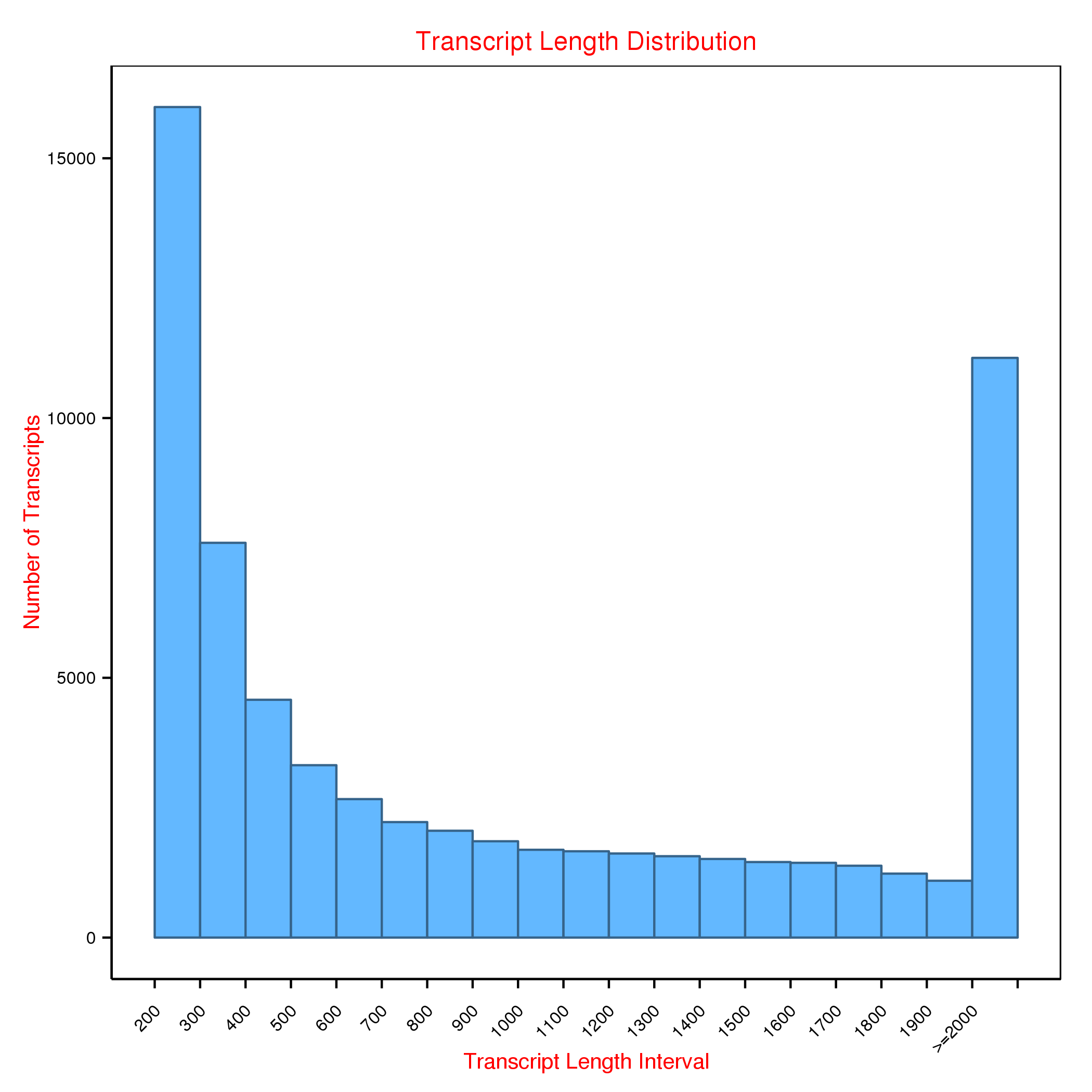


B
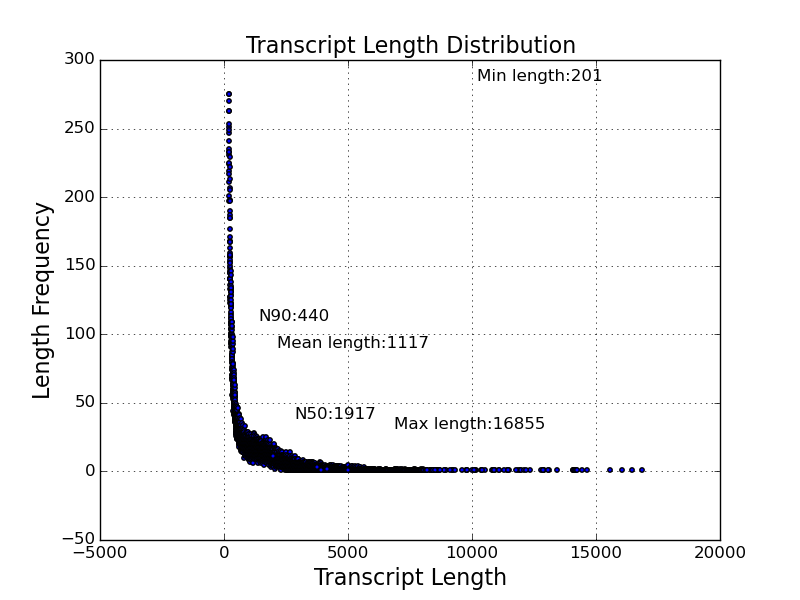


C
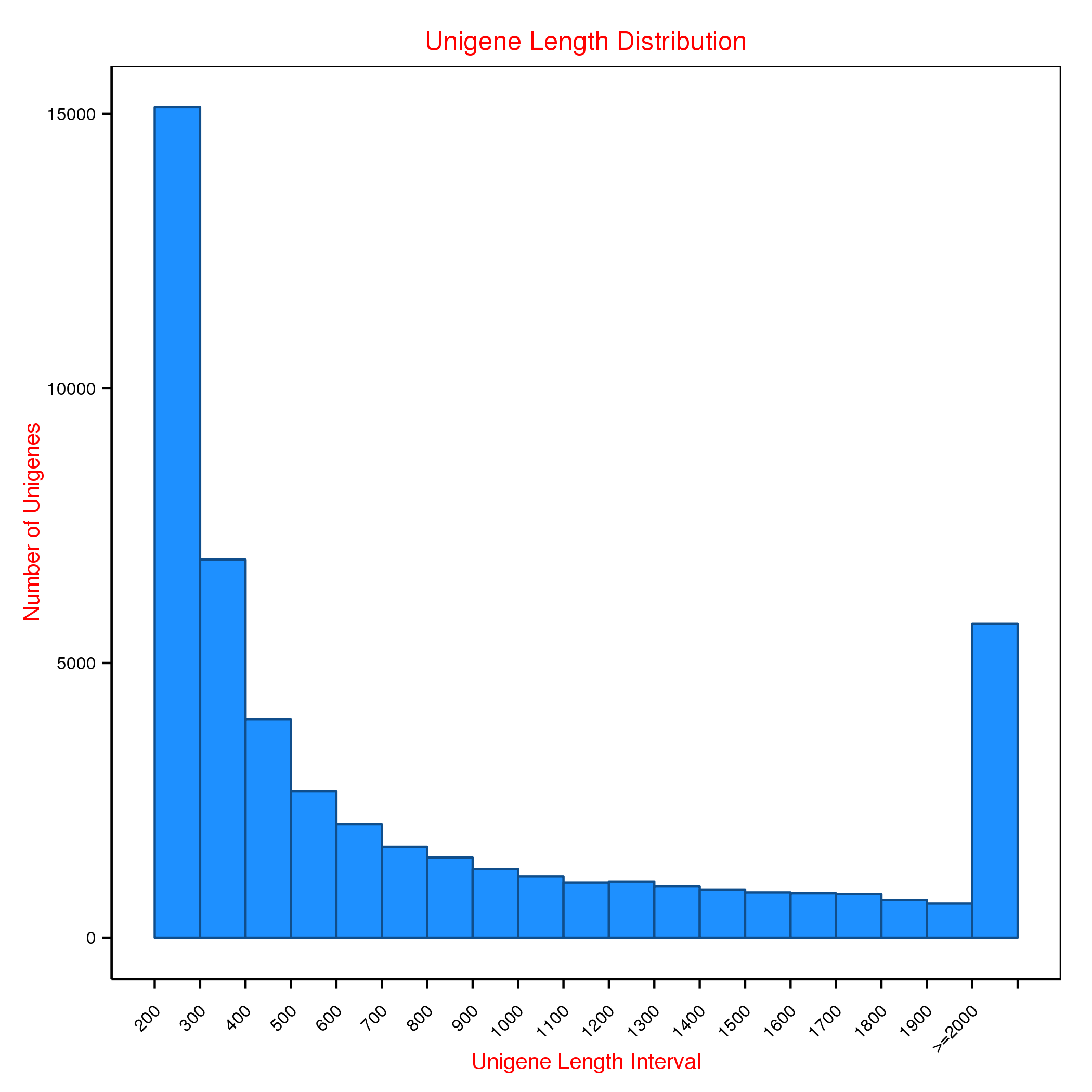


D
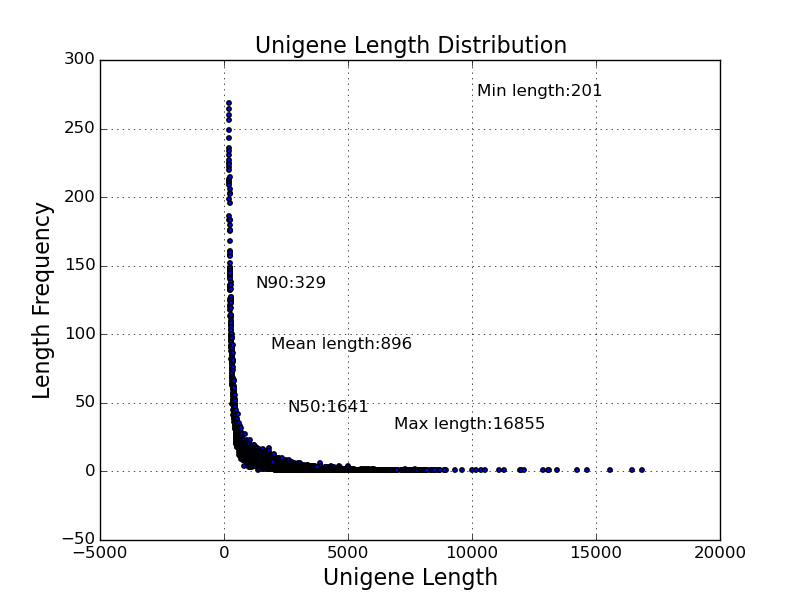


E
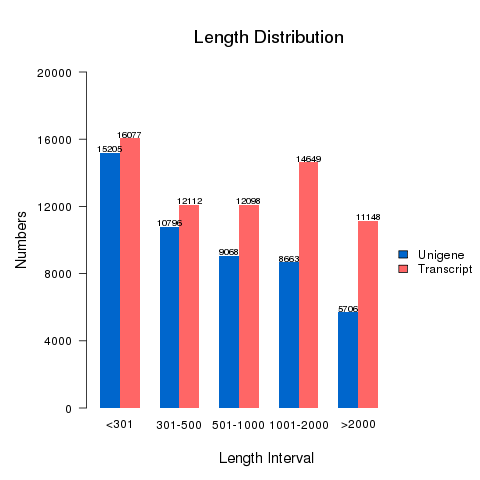


**Fig. S3** Lengths distribution of assembled of transcripts (A, B, E) and unigenes (C, D, E).
